# Quantifying Left Atrial Size in the Context of Atrial Fibrillation Ablation: Which Echocardiographic Method Correlates to Outcome of Pulmonary Venous Isolation?

**DOI:** 10.3390/jpm11090913

**Published:** 2021-09-13

**Authors:** Patrick Leitz, Lena Marie Stebel, Christian Andresen, Christian Ellermann, Fatih Güner, Florian Reinke, Simon Kochhäuser, Gerrit Frommeyer, Julia Köbe, Kristina Wasmer, Philipp S. Lange, Stefan Orwat, Lars Eckardt, Dirk G. Dechering

**Affiliations:** 1 Department of Cardiology II—Electrophysiology, University Hospital Muenster, Cardiol, Albert-Schweitzer-Campus 1, A1, 48149 Muenster, Germany; lenastebel@uni-muenster.de (L.M.S.); christian.andresen@ukmuenster.de (C.A.); christian.ellermann@ukmuenster.de (C.E.); fatih.guener@ukmuenster.de (F.G.); florian.reinke@ukmuenster.de (F.R.); simon.kochhaeuser@ukmuenster.de (S.K.); gerrit.frommeyer@ukmuenster.de (G.F.); julia.koebe@ukmuenster.de (J.K.); wasmerk@ukmuenster.de (K.W.); philippsebastian.lange@ukmuenster.de (P.S.L.); lars.eckardt@ukmuenster.de (L.E.); dirk.dechering@niels-stensen-kliniken.de (D.G.D.); 2 Department of Cardiology III—Adult Congenital and Valvular Heart Disease, University Hospital Muenster, Cardiol, Albert-Schweitzer-Campus 1, A1, 48149 Muenster, Germany; Stefan.orwat@ukmuenster.de

**Keywords:** ablation, atrial fibrillation, left atrium, echocardiography

## Abstract

Introduction: Multiple studies have shown that left atrial (LA) enlargement is a strong predictor of poor outcome after catheter ablation of atrial fibrillation (AF). LA size is commonly approximated as the diameter in the parasternal long axis. It remains unknown whether more precise echocardiographic measurements of LA size allow for better correlation with outcome after pulmonary vein isolation (PVI). Methods and results: We performed a retrospective study of 131 consecutive patients (43 females, 60% paroxysmal AF, mean CHA2DS2-Vasc score 1.6, mean age 61 ± 12 years) referred for PVI. Measurements of the LA were carried out by a single observer in transthoracic echocardiograms (TTE) performed prior to ablation. We calculated diameter of the LA in the parasternal long axis (PLAX), LA area in the 2- as well as 4-Chamber (CH) view. LA volume was computed using the disc summation technique (LAV) and indexed to body surface area (LAVI). Procedural and follow-up data were gathered from a prospective AF database. Ablation was performed exclusively using the second generation cryoballoon by the same operators. Follow-up visits at 3, 6 and 12 months showed freedom from AF in 76%, 73% and 73% respectively. Mean values of LA calculations were LAPLAX: 37.9 mm ± 6.3 mm, 2CH area: 22.5 cm^2^ ± 6.7 cm^2^, 4CH area: 21.4 cm^2^ ± 5.5 cm^2^, LAV: 73.7 mL ± 26.1 mL and LAVI: 36.2 mL/m^2^ ± 12.7 mL/m^2^, respectively. C statistic revealed the best concordance of LAVI with outcome after 12 months (C = 0.67), LAV also exhibited a satisfactory value (C = 0.61) in comparison to surfaces in 2CH (C = 0.59) and 4CH (C = 0.57). PLAX showed the worst correlation (C = 0.51). Additionally, different binary logistic regression models identified three independent predictors of AF outcome after cryoballoon PVI: gender (OR = 0.95 per year; *p* = 0.01); LAV (OR = 1.3/10mL; *p* = 0.02) and LAVI (OR = 1.58/10 mL/m^2^; *p* = 0.02). In all models, PLAX and area measurements were not predictive. Conclusions: Our data add further to evidence that LA size lends itself well as a predictor of PVI outcome. LAVI and LAV were independently predictive of rhythm outcome after PVI. This did not hold true for more commonly used measurements, such as PLAX diameter and surfaces of the LA, irrespective of the view chosen.

## 1. Introduction

Atrial fibrillation (AF) is the most common sustained arrhythmia and can be associated with debilitating symptoms. Pulmonary vein isolation (PVI), as reflected in current guidelines [1], is the most effective and therefore increasingly established therapeutic option for patients with symptomatic AF. A multitude of factors have been associated with a negative influence on outcome after PVI and might be used for patient selection. The AFFIRM trial showed LA enlargement to be associated with higher rates of AF recurrences after spontaneous, pharmacological, or electrical cardioversion [2]. Further, in patients who received a single PVI, meta-analysis showed LA dilation to be associated with a worse outcome, especially in extended follow-up [3]. With the widespread availability of echocardiography, using echocardiographically determined LA size for patient selection seems enticing.

Guidelines describe several methods to assess LA size in echocardiography with different degrees of accuracy. Although a correlation between LA size and PVI outcome has been reported in several studies, it remains unknown if more accurate measurements of LA size are stronger predictors of treatment success after PVI.

## 2. Methods

### 2.1. Study Design

The present study is in accordance with regional and institutional ethics guidelines. Retrospective data collection was approved by the local ethics committee (Ethik-Kommission der Ärztekammer Westfalen-Lippe und der Westfälischen Wilhelms-Universität, file: 2018-186-f-S), and patients gave informed consent. In our prospective database of AF ablation patients, we performed a retrospective analysis of consecutive patients who were referred to our centre for catheter ablation. A total of 131 consecutive patients were included in the study. In order to avoid bias, we opted to only include ablations using the 2nd generation Medtronic cryoballoon, no additional catheters were used in any of the included patients. No further ablations were performed.

### 2.2. Echocardiography

**Figure 1:**
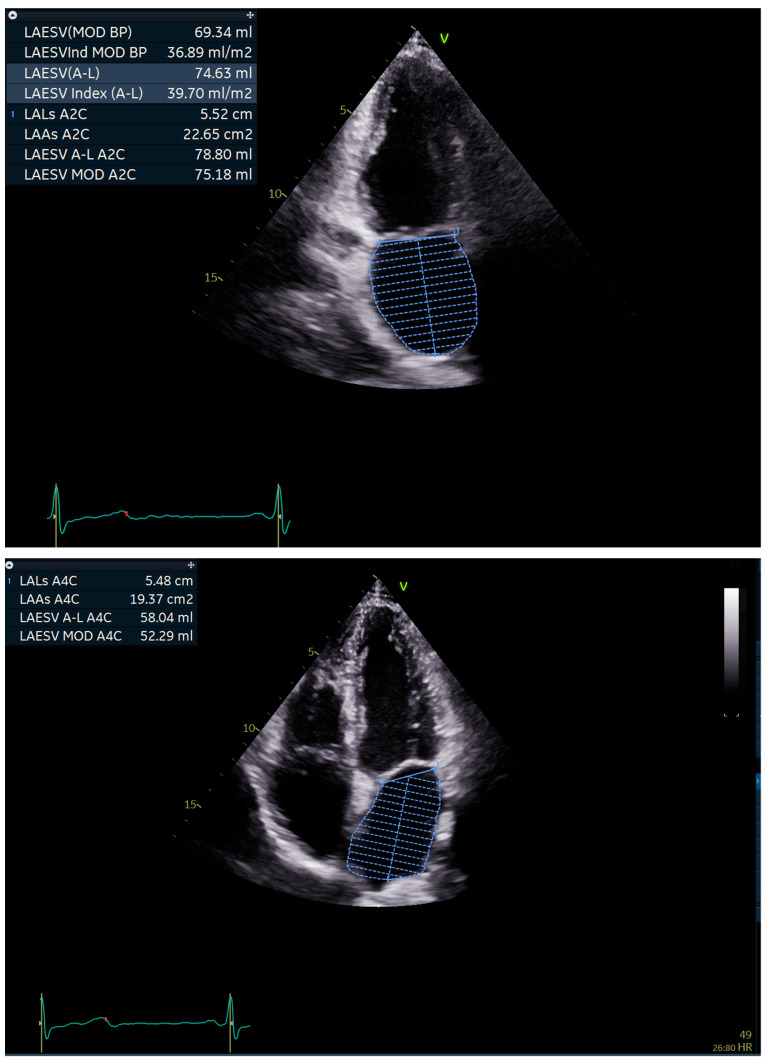
2 Chamber and 4 Chamber apical views with measurements performed at end-systole.

Prior to ablation, all patients received a complete transthoracic as well as a transoesophageal echocardiography. The left atrium was assessed exclusively by transthoracic echocardiography (TTE). The exams were performed using a commercially available ultrasound system (Vivid E95, General Electric Vingmed, Milwaukee, Wisconsin, USA). To keep data as close to clinical practice as possible, measurements were carried out with standard views and no additional acquisitions were used to account for the shift in longitudinal axes of the left ventricle and left atrium, even if they were available. Interpretation of the echocardiograms were performed blinded to the results of the follow-up. Measurements of the LA were performed by one operator (to eliminate inter-observer variability) using Image Arena^®^ (TomTec Imaging Systems GmbH, Philips, Unterschleissheim, Germany) in accordance with the recommendations of the American Society of Echocardiography [4]. In short, in 2 Chamber and 4 Chamber apical view area measurements were performed at end-systole, on the frame just prior to mitral valve opening. The inner border of the LA was traced, excluding the LA appendage, the area under the mitral valve annulus (the tracing was closed by a straight line connecting the opposite sides of the annulus at the valve level) and the inlet of the pulmonary veins (see Figure 1)

Linear measurements were obtained exclusively using 2D. Anterior–posterior measurements were performed in the parasternal long axis (PLAX) and surfaces measured in 2 Chamber, as well as 4 Chamber views. Volumetric measurements were calculated using the disk summation technique with LA areas of 2 Chamber and 4 Chamber views. Volumes were then indexed to the body surface area (LAVI). Body surface area was calculated using the Mosteller formula:

BSA (m²) = ([Height (cm) × Weight (kg)]/ 3600)½

### 2.3. Ablation Procedure

PVI was performed in all patients using the 2nd generation cryoballoon by four different experienced operators each having performed >500 PVI using the cryoballoon. Ablation with the cryoballoon as performed at our center has previously been described in detail elsewhere [5], in short: a steerable decapolar catheter (Lifewire, St. Jude Medical, Paul, MN, USA) was placed in the coronary sinus. A single transseptal puncture (TSP) was performed under fluoroscopic imaging and continuous pressure monitoring using a long, non-steerable 8 Fr sheath (SL1, St. Jude Medical). After access to the left atrium was gained, a weight-adjusted Heparin bolus was applied. From this point onwards activated clotting time (ACT) was controlled every 30 min and additional heparin was applied with a target ACT of > 300 s. The SL1-sheath was then changed to a steerable 12 Fr sheath (Flex Cath Advance; Medtronic Inc., Minneapolis, MN, USA). A single multipurpose catheter (MP1SH, Boston Scientific Inc., Natick, MA, USA) was introduced to the left atrium and pulmonary veins (PV) were visualized selectively using nonionic contrast media (Ultravist 370, Bayer, Germany). For ablation, we exclusively used the 28 mm 2nd generation cryoballoon catheter with the Achieve mapping catheter (Medtronic Inc.). Occlusion of the PV with the always fully inflated balloon was tested by injecting boli of contrasting agent. Prior to isolation of the right PV, the decapolar catheter was repositioned to the superior vena cava for phrenic nerve stimulation. A single freeze in each vein of 180–240 s was aimed, with the duration depending on time to isolation, the quality of occlusion and nadir temperature. Additional freezes were seldom used and delivered at the operators’ discretion. In the right superior and inferior veins freezes were delivered under continuous stimulation of the phrenic nerve with a cycle length of 800 ms. If temperatures fell below −60 °C, or if phrenic nerve capture was lost, the freeze was immediately aborted. Complete electrical isolation of the PVs was confirmed using the Achieve mapping catheter in sinus rhythm and during differential pacing maneuvers. At the end of the procedure, all vein isolations were again verified in sinus rhythm.

### 2.4. Follow-Up

All patients underwent scheduled visits after 3, 6 and 12 months in our outpatient clinic for follow-up. Furthermore, patients were instructed to present at our emergency department if symptomatic recurrences of atrial fibrillation occurred. Endpoint of the follow-up were symptomatic recurrences of sustained atrial fibrillation diagnosed by ECG or typical symptoms.

### 2.5. Statistical Analysis

All statistics were performed using SPSS 25.0 (IBM Corporation, Armonk, New York, USA). Continuous variables are shown as mean ± SD. The endpoint was freedom of symptomatic AF after a single PVI with the cryoballoon with a follow-up censored at 3, 6 and 12-month post PVI. Variables were compared between groups using Chi Square or Mann–Whitney tests, as applicable. We then proceeded to perform a multistep logistic regression. Variables with either univariate association or with previously known clinical correlation with PVI outcome were included along with one of the LA size measurements (to account for variance inflation factor). A two-sided *p*-value of 0.05 was considered statistically significant. Variables with a *p*-value < 0.10 in univariate regression were retained and entered in multivariate binary logistic regression, with one of the LA size determinations being forced into each model. To quantify discrimination of each method of measurement, C-statistics were calculated, where a value of 1.0 indicates perfect discrimination and a value of 0.5 is noninformative.

## 3. Results

### 3.1. Patient Characteristics

A total of 131 Patients (88 males, 43 female) were included in the study, the mean age was 60 ± 12 years. 60% of patients had paroxysmal AF, mean CHA_2_DS_2_-Vasc score was 1.6 ± 1.3. All patients were highly symptomatic (mean EHRA score 2.5 ± 0.4). Further characteristics are depicted in Table 1.

### 3.2. Procedural Findings

Mean procedural duration, defined as time from the patient entering and leaving the electrophysiology laboratory was 112 ± 28 min. The mean dose area product was 1469 Gy*cm^2^. Mean nadir temperature in left superior pulmonary vein (LSPV) was −47 ± 6 °C, in the left inferior pulmonary vein (LIPV) −44 ± 8 °C, in the right inferior pulmonary vein (RIPV) −45 ± 7°C, and in the right superior pulmonary vein (RSPV) was −48 ± 7 °C. Acute PVI of all 4 veins was achieved in 100% of the patients. None of the patients presented anatomic anomalies of the pulmonary veins.

### 3.3. Follow-Up

Follow-up visits at 3, 6 and 12 months showed 76%, 73% and 73% of patients without symptomatic AF recurrence. After 12 months follow-up, 77% of patients were paroxysmal AF recurrence-free in comparison to 68% of patients with persistent AF. In patients free from AF after 3 months, all antiarrhythmic drugs were stopped.

### 3.4. Echocardiography as Outcome Predictor

**Figure 2:**
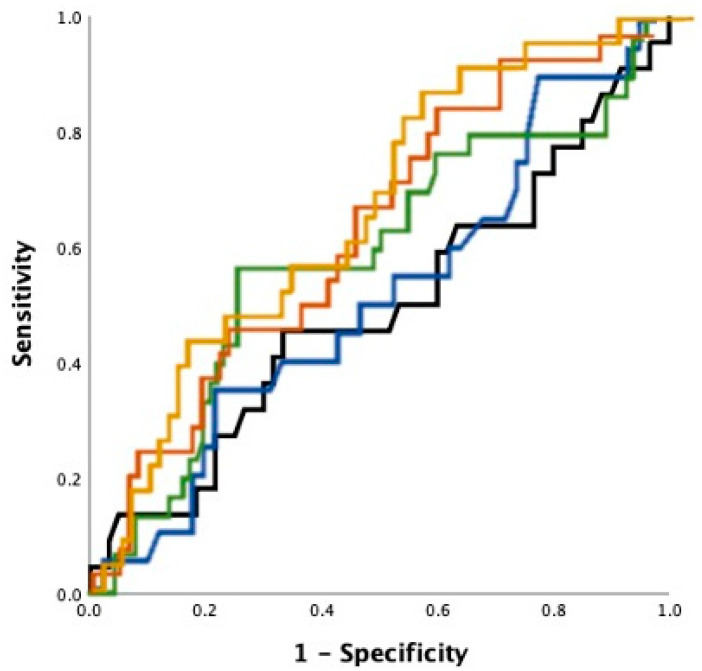
Receiver operating characteristic curve, for LAPLAX (blue), LA area in 2CH view (black) and 4CH view (green), LAV (brown) and LAVI (gold).

The vast majority showed a preserved left ventricular ejection fraction (mean 57% ± 10). Echocardiographic assessment of the LA revealed mean LAPLAX of 37.9 mm ± 6.3 mm (SD), mean LA areas of 22.5 cm^2^ ± 6.7 cm^2^ (2-Chamber (CH)), 21.4 cm^2^ ± 5.5 cm^2^ (4CH) and LAV and LAVI with mean values of 73.7 mL ± 26.1 mL and 36.2 mL/m^2^ ± 12.7 mL/m^2^, respectively. Results from binary logistic regression analysis are summarized in Table 2. Multivariate analysis consistently identified three independent predictors of PVI outcome with the cryoballoon: gender (OR = 0.95; *p* = 0.01); LAV (OR = 1.3/10mL; *p* = 0.02) *or* LAVI (OR = 1.58/10 mL/m^2^; *p* = 0.02), depending on the model used. C statistic revealed the best discriminatory abilities of LAV (*C* = 0.61) and LAVI (*C* = 0.67) with outcome after 12 months. A LAVI of 50 mL/m^2^ for example showed a sensitivity of 83% and a specificity of 74%. PLAX correlation with outcome was poor (*C* = 0.51). Receiver operating characteristic curves are depicted in Figure 2.

## 4. Discussion

To the best of our knowledge, we present the first study directly comparing five different 2d-echocardiographic methods of LA size determination and their ability to determine PVI outcome.

We could demonstrate that only more sophisticated LA measures, such as LAV and LAVI, were independent predictors of recurrence of AF after PVI in our mixed paroxysmal/persistent all-comer AF cohort. Left atrial volumes indexed to body surface showed the strongest correlation with outcome after catheter ablation, while PLAX showed no correlation. After a single PVI in our mixed collective of patients with paroxysmal and persistent AF, 73% were free from symptomatic AF after 1 year, which is in line with published data [6].

Most clinicians use transthoracic echocardiography (TTE) or multi-slice computed tomography scan (CT) to measure LA size. Magnetic resonance imaging (MRI) seems enticing with promises of high resolution and no radiation; however, in many countries MRI is not easily available and/or reimbursed in the context of PVI. Reimbursement issues are also seen in CT scans in many countries. TTE remains the safest, most economical, readily available and least time-consuming modality. However, the quality of the method suffers in certain patients, e.g., morbidly obese patients or patients with serious obstructive pulmonary disease.

Further accuracy in determining LAV by TTE may be achieved by performing 3D derived measurements [7]. LA strain is also of interest in determining atrial myopathy. Both these methods are however not part of the daily clinical practice in most low to medium volume centres around the world. We therefore limited the presented analysis to 2d measurements.

Most of the current literature on correlation of PVI outcome and LA size use PLAX to quantify LA and the available data are inconsistent. In a meta-analysis of predictors of AF recurrence after radio frequency ablation, for example, Balk et al. [8] analyzed PLAX and found it to be an independent predictor of PVI outcome in only four of 20 studies. In our presented cohort, PLAX also showed no valid correlation with freedom of AF after PVI. On the other hand, Zhuang et al. [3] examined 22 studies with a total of 3750 patients undergoing a single radio frequency PVI in their meta-analysis on predictors of PVI outcome. The authors found PLAX to be a strong predictor of AF recurrence. The conflicting findings may be interpreted as a result of the poor correlation of PLAX to the true LA size. As dilation of the LA is not symmetrical and is mainly happening in superior–inferior and medial–lateral direction, the two-dimensional measurement may struggle to reflect the actual size of the LA. Furthermore, left atrial [9] size is significantly linked to body surface area as well as body mass index in non-AF populations. Without taking into account patients’ size and weight, LA measurements may result in pathological interpretation of physiological dimensions.

Using a second plane to calculate volumes helps better approximate true LA size as reflected in our data where LAV and LAVI showed to be independent predictors of sole PVI outcome. Several studies were able to show in patients with lone AF, LA enlargement to be progressive over the course of the arrhythmia [10,11,12]. In comparison to patients with persistent AF, patients with paroxysmal AF with low arrhythmia burden show no to little LA enlargement and subsequent LA substrate, and are known to show superior outcomes after PVI [13]. In our study, no additional substrate-based ablation was performed, explaining why the most accurate LA size determination shows the best outcome correlation.

Available research on LA volumes as a predictor for AF recurrences after PVI is scarce and heterogenous. Njoku et al. [14] performed a meta-analysis of the most available data. LA size was assessed by either transthoracic echocardiogram (TTE), cardiac computed tomography (CT), or magnetic resonance imaging (MRI). Eleven studies with a total of 1559 subjects were included to evaluate the effect of LAV on PVI outcome. The authors were able to show LAV to be an independent predictor of outcome. In a second step, the authors proceeded to also analyze the difference in left atrial volume index in patients with and without AF recurrence. In nine studies with a total of 1425 patients, a large LAVI was associated with poor PVI outcome.

Univariate analysis of the presented data also revealed patients’ gender and weight at the time of PVI to influence outcome. Multistep logistic regression retained patients’ gender as an independent predictor of PVI outcome with the cryoballoon, with females showing less satisfactory outcomes. Several different possible explanations of poor PVI outcome in females have been reported in the past. For one, a sub-analysis of the AFFIRM trial showed that LA diameter enlargement is associated with the female sex. Further, studies aiming to examine gender specific outcomes of AF ablation showed women were less likely to be referred to catheter ablation and showed a longer history of AF at the time of PVI [15,16]. All these factors may contribute to the observed worse outcome after PVI in females.

## 5. Limitations

We did not perform continuous ECG monitoring, as a result some asymptomatic recurrences of AF may have been missed. Further, because patients with a relatively normal BMI were included in the present study, the findings may not be generalizable. For example, in overweight and obese patients, indexing the LA size to BSA may overcorrect LA size.

## 6. Conclusions

Our findings emphasize the role of LA size in patient selection for pulmonary vein isolation. However, left atrial diameter and two-dimensional areas proved to be of little to no help in predicting freedom of AF after PVI. Using more sophisticated methods of measurement, such as LAV and LAVI, showed significant correlation with cryoballoon PVI outcome.

## Figures and Tables

**Table 1:** Baseline characteristics of patient population.

	Complete Collective	Patients Free from AF at 12 Month FU (*n* = 93)	Patients with AF Recurrence at 12 Month FU (*n* = 38)	*p*-Values
Age (Years)	61 ± 12	60 ± 12	62 ± 9	0.5
Persistent AF (%)	40%	30%	45%	0.38
CHA_2_DS_2_-Vasc Score	1.6 ± 1.3	1.6 ± 1.4	1.8 ± 1.2	0.29
Diabetes (%)	8%	10%	3%	0.27
Arterial Hypertension (%)	51%	52%	58%	0.59
Prior Stroke (%)	9%	8%	3%	0.34
BMI (kg/m^2^)	26 ± 3	27 ± 3	25 ± 2	0.1
EHRA Class	2.5 ± 0.5	2.5 ± 0.5	2.7 ± 0.4	0.01
Female gender	*n* = 48	*n* = 26	*n* = 22	0.01

**Table 2:** Statistical analysis: Univariate and multivariate analysis of LA measurements.

	Univariate OR	Univariate *p*	Multivariate OR	Multivariate *p*	C Statistic
PLAX	1.05/10 cm	0.91	1.7/10 cm	0.2	0.51
Area 2CH	1.4/10 cm^2^	0.28	1.9/10 cm^2^	0.05	0.59
Area 4CH	1.3/10 cm^2^	0.48	1.9/10 cm^2^	0.07	0.57
LAV	1.1/10ml	0.19	1.3/10ml	0.02	0.61
LAVI	1.4/10 mL/m^2^	0.03	1.58/10 mL/m^2^	0.02	0.67
